# Natural Product Celastrol Destabilizes Tubulin Heterodimer and Facilitates Mitotic Cell Death Triggered by Microtubule-Targeting Anti-Cancer Drugs

**DOI:** 10.1371/journal.pone.0010318

**Published:** 2010-04-23

**Authors:** Hakryul Jo, Fabien Loison, Hidenori Hattori, Leslie E. Silberstein, Hongtao Yu, Hongbo R. Luo

**Affiliations:** 1 Department of Pathology, Dana-Farber/Harvard Cancer Center, Harvard Medical School, Boston, Massachusetts, United States of America; 2 Department of Pharmacology, Howard Hughes Medical Institute, University of Texas Southwestern Medical Center, Dallas, Texas, United States of America; Dresden University of Technology, Germany

## Abstract

**Background:**

Microtubule drugs are effective anti-cancer agents, primarily due to their ability to induce mitotic arrest and subsequent cell death. However, some cancer cells are intrinsically resistant or acquire a resistance. Lack of apoptosis following mitotic arrest is thought to contribute to drug resistance that limits the efficacy of the microtubule-targeting anti-cancer drugs. Genetic or pharmacological agents that selectively facilitate the apoptosis of mitotic arrested cells present opportunities to strengthen the therapeutic efficacy.

**Methodology and Principal Findings:**

We report a natural product Celastrol targets tubulin and facilitates mitotic cell death caused by microtubule drugs. First, in a small molecule screening effort, we identify Celastrol as an inhibitor of neutrophil chemotaxis. Subsequent time-lapse imaging analyses reveal that inhibition of microtubule-mediated cellular processes, including cell migration and mitotic chromosome alignment, is the earliest events affected by Celastrol. Disorganization, not depolymerization, of mitotic spindles appears responsible for mitotic defects. Celastrol directly affects the biochemical properties of tubulin heterodimer *in vitro* and reduces its protein level *in vivo*. At the cellular level, Celastrol induces a synergistic apoptosis when combined with conventional microtubule-targeting drugs and manifests an efficacy toward Taxol-resistant cancer cells. Finally, by time-lapse imaging and tracking of microtubule drug-treated cells, we show that Celastrol preferentially induces apoptosis of mitotic arrested cells in a caspase-dependent manner. This selective effect is not due to inhibition of general cell survival pathways or mitotic kinases that have been shown to enhance microtubule drug-induced cell death.

**Conclusions and Significance:**

We provide evidence for new cellular pathways that, when perturbed, selectively induce the apoptosis of mitotic arrested cancer cells, identifying a potential new strategy to enhance the therapeutic efficacy of conventional microtubule-targeting anti-cancer drugs.

## Introduction

Microtubules (MTs), filamentous polymers of alpha- and beta-tubulin heterodimer, are essential cytoskeleton structures that control fundamental cellular processes, such as cell division, migration, intracellular transport and signaling (for review[1]). MTs are dynamic in nature and constantly undergo phases of shrinkage and re-growth, a process known as ‘dynamic instability’. This dynamic property underlies the vast majority of MT-based cellular processes [1]. The overall dynamics of MTs vary in different cell types and cellular contexts and are regulated at the multiple levels, including post-translational modifications of tubulin itself and interactions with a wide variety of MT binding proteins [2], [3], [4], [5], [6].

Under physiological conditions, the most dramatic reorganization of MTs occurs at the onset of mitosis when the interphase MTs are depolymerized and repolymerized to form mitotic spindles. The spatio-temporal assembly and dynamic behavior of mitotic spindles are critically important for proper alignment and segregation of the duplicated chromosomes, failure of which leads to cell death or genomic instability (for review [7]). The mitotic spindles are particularly sensitive to various natural and synthetic MT drugs that impair their assembly and functions, leading to mitotic arrest and subsequent cell death. Due to this activity, MT drugs are most widely used to treat human cancers. However, some cancer cells are intrinsically resistant and the drug-exposed cancer cells often acquire a resistance.

Discovery and exploitation of structurally different new types of antimicrotubule agents may overcome such problem. For example, the clinical utility of structurally different MT stabilizers to overcome the Taxol-resistance has been reported [8], [9], [10]. The complementary approaches may include a combined inhibition with other cellular factors, such as mitotic kinases and motor proteins [11]. Nevertheless, given the essential functions of MTs in various cellular processes, some degrees of cytotoxicity to normal cells appear inevitable.

It has been reported that the relative resistance of various human cancer cell lines to common antimitotic agents is correlated with lack of cell death rather than mitotic arrest [12]. This finding is consistent with both clinical observations and animal model experiments that identified the degree of cell death following Taxol treatment determined the overall outcome of its efficacy [13], [14]. Therefore, genetic or pharmacological agents that selectively facilitate apoptosis of mitotic arrested cells (i.e. proliferating cancer cells) present opportunities to strengthen the efficacy of MT drugs, with little impact on the drug-exposed interphase cells (i.e. non-dividing normal cells).

In the course of small molecule screening effort (unpublished result), we identified a natural product Celastrol, traditionally known for its anti-inflammatory and anti-cancer activities, as an inhibitor of neutrophil chemotaxis. By employing a series of experiments which include the time-lapse imaging of cell migration and chromosome alignment as well as biochemical and cell biological approaches, we revealed that inhibition of MT functions was one of the earliest cellular events affected by the novel tubulin-targeting activity of Celastrol. We further demonstrated that this unique activity could be exploited to induce apoptosis of Taxol-resistant cancer cells, and to selectively facilitate the mitotic cell death of MT drug-arrested cancer cells. These findings provide a molecular explanation for the anti-inflammatory and anti-cancer activity of Celastrol, and present a potential strategy to enhance the mitotic cell death induced by conventional microtubule-targeting anti-cancer drugs.

## Materials and Methods

### Cell culture and generation of Taxol-resistant cell line

Both HEK293 and HeLa cell lines were obtained from American Type Culture Collection (ATCC), and were maintained in DMEM supplemented with 10% fetal bovine serum and 1% penicilin and streptomycin under 5% CO_2_.

To generate a Taxol-resistant cell line, HeLa cells stably expressing the EGFP fused to Histone 2B were generated. These cells were then plated into 60 mm-culture dish (1×10^6^) in the presence of 0.5 nM of Taxol. After 72 hours, the live cells were collected and re-plated in the presence of the same concentration of drug. This procedure was repeated at least three times for a given drug concentration. The concentration of Taxol was gradually increased to a final concentration of 5 nM. The resistant cells designated as H2B-TxR were maintained and propagated in the presence of 5 nM Taxol.

### Reagents and antibodies

Celastrol was purchased from EMD Biosciences and all other chemicals unless specified were from Sigma Aldrich and Tocris Bioscience. Mouse monoclonal antibodies for gamma-Tubulin (T3320), alpha-Tubulin (T6199), and beta-Tubulin (T4026) were from Sigma Aldrich; Rabbit polyclonal antibodies for alpha Tubulin (ab18251) and beta-Tubulin (ab6046) were from Abcam. The HRP-conjugated anti-rabbit and anti-mouse secondary antibodies were from Amersham Biosciences; all other antibodies were purchased from Cell Signaling Technology.

### Neutrophil purification and EZ-taxiscan chemotaxis assay

Human peripheral blood neutrophils were purified as previously described [15]. The EZ-taxiscan chamber (Effector Cell Institute, Tokyo, Japan) was assembled with a 260 µm wide ×4 µm thick silicon chip according to the manufacturer instruction. The inhibitor-treated neutrophils (3×10^6^/ml) were mixed in RPMI (0.1% BSA) and loaded to the lower chamber (3000 cells per well). One microliter of fMLP (100 nM final) was added to the upper chamber. The migrating neutrophils toward the upper chamber were imaged every 30 seconds for 20 minutes. The movie was analyzed using DIAS software (Solltech, Oakdale, IA) to calculate the speed.

#### Western blot and immnunostaining

Preparation of total cell lysates, Western blot, and other standard molecular biological techniques were essentially the same as described previously [16]. For the analysis of mitotic nuclei, cells were fixed in the presence of 3% PFA (pre-warmed) for 5 minutes before DAPI staining. For immunostaining of microtubules, cells were plated into a 35 mm-glass bottom dish (MatTek Corp.) and fixed for 5 minutes in pre-chilled (−20°C) methanol. After washing three times in PBS-Triton X-100 (0.05%), the fixed cells were permeabilized for 30 minutes in 5% normal goat serum containing 0.3% Triton X-100. The diluted antibody (1∶2000 for primary and 1∶1000 for secondary antibody) in the same solution was added and incubated for 3 hours at room temperature. After washing three times in PBS-Triton X-100, the Alexa fluor dye-conjugated secondary antibody was added and incubated for 1 hour at room temperature. The staining was visualized under the fluorescent microscope (Olympus IX71) and the image was taken using the 100 X objective lens. To examine the defects of the positioning of two centrosomes with respect to substratum in Celastrol-treated mitotic cells, two images with different focal planes, focused on the gamma-tubulin staining of each pole, were taken along with alpha-tubulin staining in the spindles (**Figure S1B**).

### Biochemical assays for tubulins

The exponentially growing HEK293 cells were collected and washed once in PBS. The cell pellet was frozen on dry ice for 15 minutes and lysed in the assay buffer containing 0.3% CHAPS, 200 µM GTP, and the protease inhibitor cocktail (Sigma Alderich) in PBS. The lysate was kept on dry ice for 15 minutes and thawed at room temperature. Once thawed, the cell lysate was immediately centrifuged at 14,000 rpm for 5 minutes at 4 degree. Only the freshly prepared cell lysate (2–4 µg/ml) was used in *in vitro* oligomerization assay, typically in a 50 µl reaction volume. After pre-incubation in the presence of drug for 10 minutes on ice, the lysate was incubated at 37°C for 15 to 60 minutes. The reaction was stopped by adding an equal volume of 2 X LDS buffer and boiled for 5 minutes prior to SDS-PAGE. For the assay with the purified tubulin (>99% from Cytoskeleton, Inc), the tubulin solution was diluted to a concentration of 10 µg/ml in the assay buffer, and the reaction was performed essentially the same as the cell lysate. For immunoprecipitation, HEK293 cells were lysed in the same buffer as above (typically 1 ml per 60 mm-culture dish). The lysate was cleared by centrifugation and incubated on ice for 10 minutes in the presence of different drugs. The polyclonal alpha tubulin antibody (ab18251, Abcam) was added (5 µg of antibody per 1 mg of protein per sample) and incubated for 1 hour in the cold room before adding the protein G/A-agarose slurry (30 µl per sample). After incubation for additional 2 hour, the immunecomplex was washed three times in the ice-cold assay buffer at 4°C before SDS-PAGE.

### Caspase activity, cell viability, and proteasome activity assay

The colorimetric caspase activity assay was carried out using the crude cell extract in the presence of caspase substrate, Ac-DEVD-pNA (Biomol International). The drug treated cells were collected and washed once in PBS. Cells were lysed on ice for 10 minutes in a buffer containing 0.1% CHAPS, 50 mM HEPES (pH 8.0), 12.5 mM NaCl, 0.1 mM EDTA, and 5 mM DTT freshly added. The lysate was centrifuged for 5 minutes at 14, 000 rpm at 4 degree. The cleared lysate (approximately 200–400 µg of protein) was mixed with the Ac-DEVD-pNA (200 µM final) in 100 µl reaction volume in a 96-well assay plate. The plate was incubated at 37°C and the enzyme activity was measured by reading the absorbance at 405 nm for every 1 hour using the plate reader (TriStar LB 941, Berthold Technologies). The cell viability was determined by MTT assay as described previously [16]. To measure proteasome activity, HEK293 cells (1×10^6^) were treated with each chemicals (5 µM) for 1 hour, and lysed on ice for 15 minutes in 200 µl of lysis buffer (20 mM TrisHCl, pH 7.5; 150 mM NaCl; 1 mM EDTA; 1 mM EGTA; 1 mM beta-Glycerophosphate; 1% Triton- X100). After clearing cell debris by centrifugation at 4 degree, the extract was subjected to proteasomal activity assay using the Proteasome-Glo™ Chymotrypsin-Like Assay (Promega).

### Time-lapse live cell imaging

For cell migration assay, HeLa cells expressing Histone H2B EGFP (HeLa-H2B) were plated into a 35-mm culture dish (4×10^5^) and cultured for 48 hours. The scratch was made using the tip in the middle of dish and washed once in pre-warmed Leibovitz's L15 medium supplemented with 0.5% FBS. After 10 minutes of healing in the same medium, the drug was added and the time-lapse movie was taken every 15 minutes for 150 minutes using the IPLab software. The migration area was determined by subtracting the area occupied by cells at time 150 minutes with that of at time 0 minute for each drug. The relative migration area was calculated by normalizing against the control (DMSO-treated) group. For mitotic chromosome alignment, HeLa-H2B cells were plated into a 35 mm-glass bottom dish at 20% confluence and cultured for 48 hours. The medium was replaced with Leibovitz's L15 medium supplemented with 0.5% FBS. Immediately following the addition of each drug, the prophase cells were identified under the fluorescent microscope and the time-lapse movie was taken every 5 minutes for 90 minutes. For apoptotic death of mitotic arrested cells, HeLa-H2B cells growing exponentially (or around 70% confluence) in 35 mm-dish were replaced with 2 ml of Leibovitz's L15 (0.5% FBS) medium containing 10 nM Vinblastine and cultured for 4 hours. After addition of each drug, the time-lapse movie was taken every 15 minutes for 4 hours. The apoptotic death of only the ‘pre-arrested’ mitotic cells, as identified by round morphology at time 0, was scored and designated as ‘mitotic death’ (see **Figure S4**). The non-mitotic cell death was defined as the adherent and flat cells (drug-exposed interphase cells) died in the next two frames (within 30 minutes). The apoptotic death of ‘newly-arrested’ mitotic cells during time-lapse imaging was not counted. To determine the effects of Celastol on mitotic cells, we synchronized and enriched mitotic HeLa-H2B cells by ‘double-thymidine block’ [17]. Briefly, cells were grown to 20–30% confluency and cultured for 19 hrs in the presence of thymidine (2 mM), and incubated for 10 hours without thymidine. The second block was carried out by culturing for 17 hours with thymidine (2 mM). Cells were washed and incubated for 5 hours without thymidine, and the medium was replaced with Leibovitz's L15 (0.5% FBS) medium. Cells were cultured for additional 4 hours before adding Celastrol, and the fate of mitotic cells were tracked by time-lapse movie.

## Results

### Celastrol inhibits cell migration

**Figure 1:**
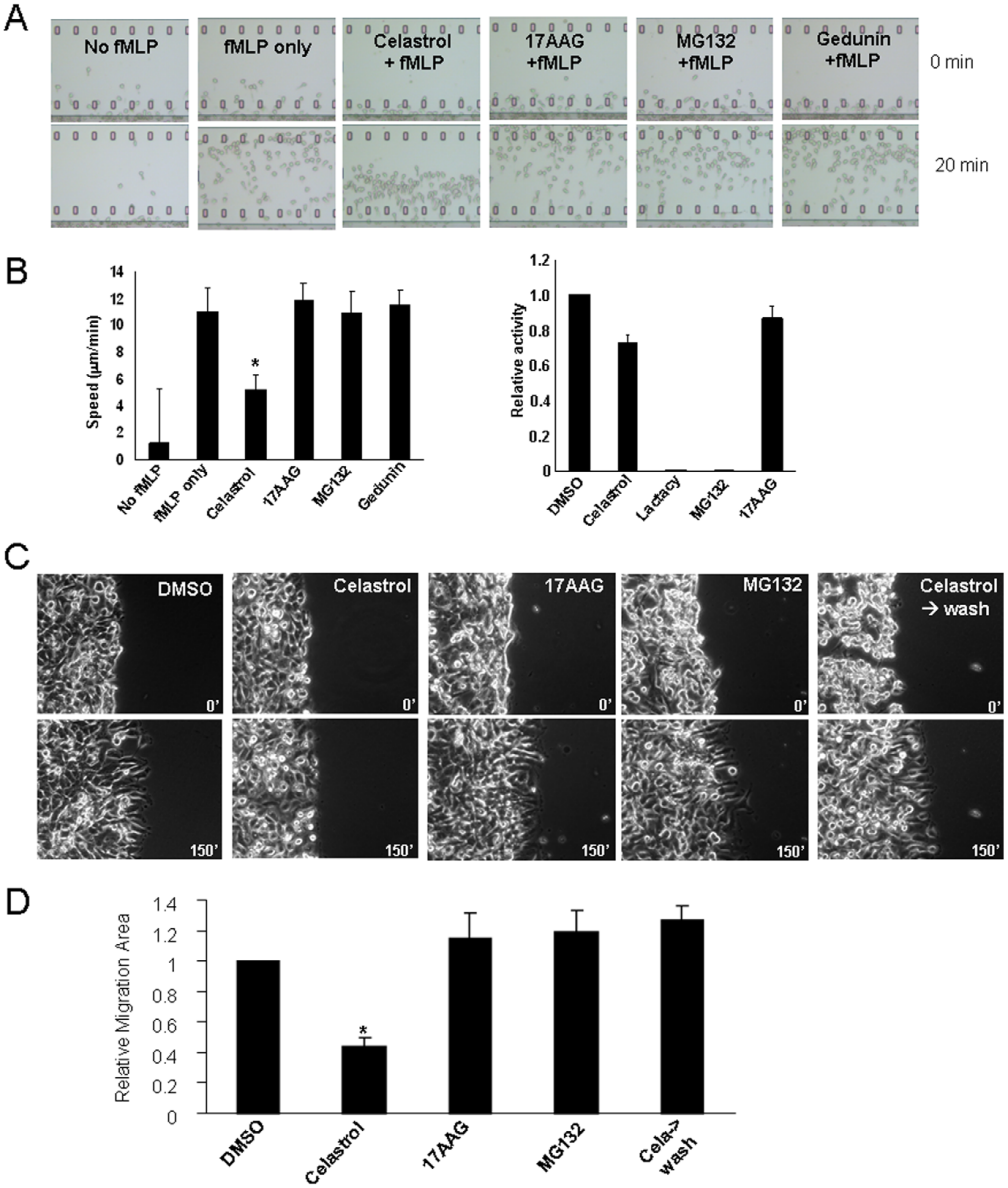
Celastrol inhibits cell migration. (**A–B**) Celastrol inhibits fMLP-induced neutrophil chemotaxis. (**A**) Freshly isolated human peripheral blood neutrophils were pre-treated with each chemical for 30 minutes, and then subjected to fMLP-mediated chemotaxis for 20 minutes using the EZtaxiscan. The first and the last images of the videos were shown for each drug. fMLP (100 nM), Celastrol (2 µM), 17AAG (4 µM), MG132 (10 µM), and Gedunin (20 µM) were used in the indicated combination. (**B**) Left panel is the quantification of the migrating speed upon each drug treatment. Right panel is the whole cell lysate assay of the proteasomal activity treated with each drug for 1 hour. The concentration of all chemicals was 5 µM. (**C–D**) Celastrol reduces epithelial cancer cell motility. (**C**) The confluent HeLa cells were scratched using a tip. After 10 minutes of recovery, each drug was added and the time-lapse images were taken every 15 minute for 150 minutes. The representative still images of the first (time 0) and the last (time 150) time points were shown. For the sample of “Celastrol-wash”, cells were pre-treated with Celastrol for 3 hour before scratching; and the time-lapse movie was taken in a drug-free medium. (**D**) Quantification of cell migration. The relative migration area over the control (DMSO-treated) was presented. * indicates p<0.05 by student T-test.

Celastrol is one of the active compounds derived from plant extracts traditionally used to treat the inflammatory symptoms [18]. Recruitment of immune cells to the site of inflammation precedes a cascade of cellular events leading to inflammatory responses, and inhibition of this recruitment can attenuate the inflammatory processes. During a small molecule screening, Celastrol was identified as an inhibitor of the fMLP-induced neutrophil chemotaxis (unpublished result). Celastrol has been shown to inhibit the proteasomal activity and modulate the heat shock protein (HSP) 90 pathway [19], [20], [21]. Therefore, we examined the effects of known inhibitors of these pathways, 17AAG for HSP90 and MG132 for proteasome, under the same experimental setting. Compared with the control cells, the Celastrol-treated neutrophils showed an approximately 50% reduction in migrating speed toward a gradient source of fMLP (**Movie S1 and S2**). However, no detectable effect was observed in the presence of other chemicals. In addition, Gedunin, which has been shown to inhibit the HSP90 pathway similar to Celastrol [20], also showed no inhibitory effect (**Figure 1A and B**). Moreover, when tested in the whole cell lysate assay, Celastrol showed a very little inhibitory activity toward proteasome (**Figure 1B**). Thus, the effect of Celastrol on neutrophil chemotaxis is not caused by the inhibition of the proteasomal activity.

Next, we explored if this finding could be extended to other cell types. A recent study showed that Celastrol inhibited TNF alpha-induced tumor cell invasion through inhibition of gene expression controlled by NF-κ B pathway [22]. However, due to a longer duration of drug exposure in this study, whether or not Celastrol directly affected the motility of epithelial cells could not be determined. Accordingly, we examined the direct effect of Celastrol on tumor cell migration using a wound healing assay. The confluent Hela cells were scratched, and time-lapse images of migration toward the wounded area were taken. Consistent with its effects on neutrophil chemotaxis, Celastrol effectively inhibited cell migration, on average 60% reduction over the time course of 150 minutes (**Movie S3 and S4**). To rule out the possibility that this inhibitory effect was due to the general cell toxicity, we pre-treated cells with Celastrol for 3 hrs. Upon removal of drug, the migration capacity of cells was fully restored, indicating this inhibitory effect was reversible and was not caused by general toxicity (**Figure 1C and D**). Similar with what was observed in neutrophils, inhibition of either proteasome or HSP90 pathway had no inhibitory effect under this experimental condition (**Figure 1C and D**). These results indicate the presence of cellular target(s) of Celastrol, insensitive to inhibition of the proteasome or HSP90 pathway.

### Celastrol impairs mitotic progression by inhibiting chromosome alignment

**Figure 2:**
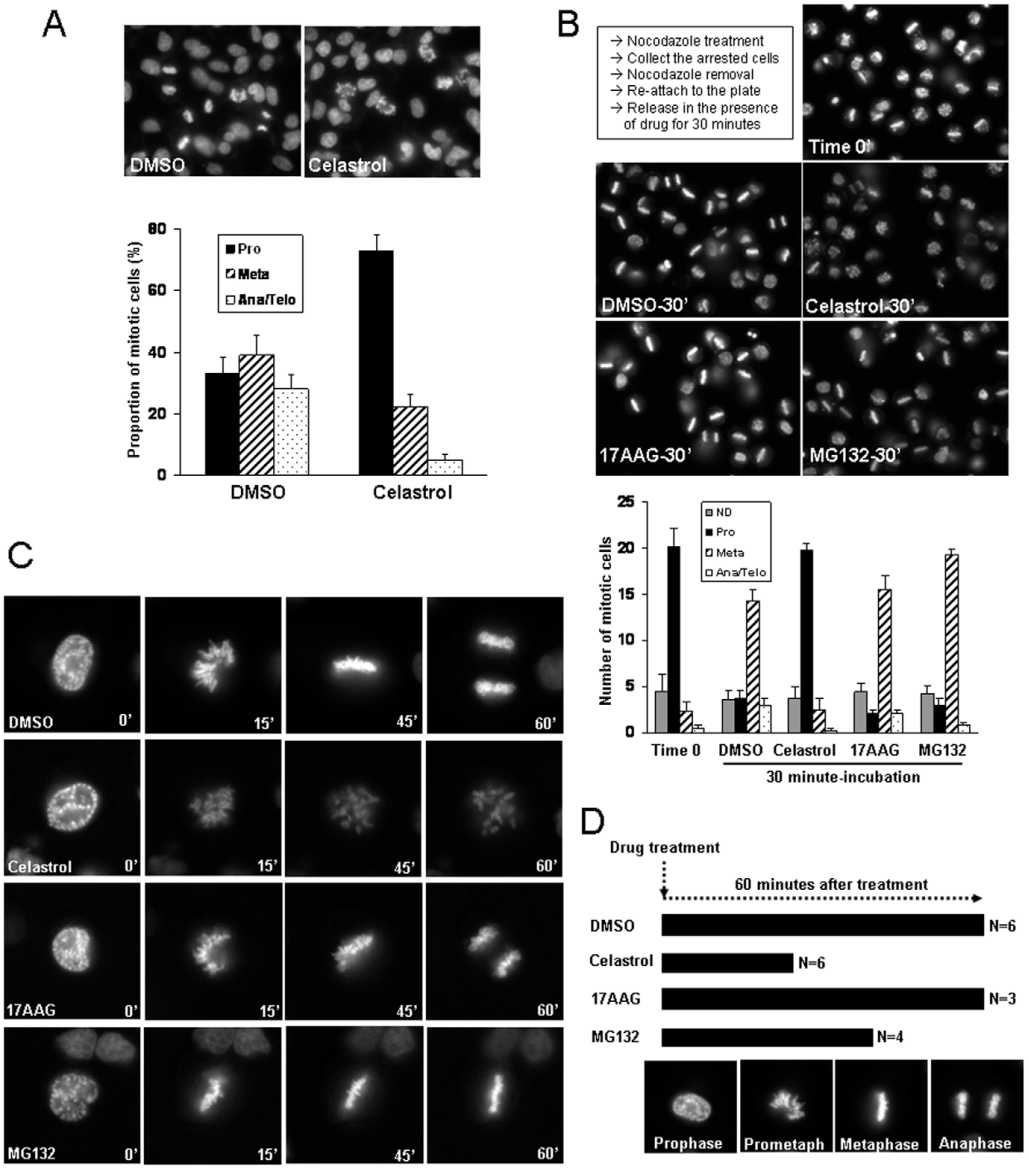
Celastrol inhibits mitotic progression and chromosome alignment. (**A**) HEK293 cells were treated with Celastrol (2 µM) for 1 hour before fixation and staining with DAPI. The mitotic cells were counted and scored according to their chromosomal morphology. The proportion of each mitotic stage over the total number of counted cells was presented. (**B**) HeLa cells were treated with Nocodazole (100 nM) for 6 hours. The arrested cells were collected by tapping the plate, washed in PBS, and replated in a serum free medium. After 15 minutes of attachment (time 0), each drug was added and incubated for 30 minutes before fixation and staining with DAPI. The average number of cells in each stage from the quadruplicates was shown. ‘ND’ indicates the non-mitotic or dead cells. (**C**) Hela cells over-expressing EGFP-H2B protein were treated with indicated drugs. Immediately following drug addition, the prophase cells were identified and the time-lapse images were taken every 5 minutes for 90 minutes. The magnified still images from the representative movies were shown. (**D**) The summary of time-lapse movies for mitotic chromosome alignment in C). The degree of mitotic progression of prophase cells in 60 minutes in the presence of each chemical was determined based on chromosomal shape, and was indicated by horizontal bar. Numbers indicate number of cells examined. The representative chromosomal shapes of control cells at different mitotic stages were shown in lower panel as a reference.

The dynamic and coordinated regulation of cytoskeleton network, such as actin filaments and microtubules, is critical for migrating cells. The inhibitory effects on cell migration in both neutrophil chemotaxis and epithelial wound-healing suggest a possibility that Celastrol may interfere with the cytoskeleton network. During mitosis, MT dynamics plays crucial roles in alignment and segregation of chromosomes. Therefore, we examined for abnormalities in the proportion of mitotic phases upon Celastrol treatment. While the DMSO-treated HeLa cells showed a similar proportion of prometaphase, metaphase, and anaphase/telophase chromosomes, over 70 percent of chromosomes in Celastrol-treated cells displayed the ‘prometaphase-like’ phenotypes (**Figure 2A**). To further confirm this result, we performed a prometaphase-arrest and release experiment (**Figure 2B**). We first arrested cells at the prometaphase by Nocodazole treatment, and the arrested cells were collected by tapping the plate. Upon removal of Nocodazole, the mitotic spindles are re-assembled, which align chromosomes to the metaphase plane for the subsequent mitotic progressions to occur. This release experiment was carried out in the presence of different chemicals to assess their effects on the mitotic spindle functions. In control group, almost half of the arrested cells have progressed to anaphase during 30 minute-release (incubation) period. However, in the presence of Celastrol, the majority of cells were arrested in the prometaphase, while inhibition of HSP90 had no effect. The proteasome inhibition by MG132 slightly accumulated cells at the metaphase (**Figure 2B**). To further validate these observations, we performed a live imaging of mitotic cells (**Figure 2C and D**). Immediately following the addition of each chemical, the prophase cells, as identified by the condensed chromatids, were imaged as they undergo chromosome alignment and segregation (**Movie S5 and S6**). In control group, the prophase cells progressed to anaphase within 60 minutes. However, in the presence of Celastrol, they failed to progress and stayed in a prometaphase-like state. Consistent with the results from the prometaphase-arrest and release experiment, inhibition of HSP90 had no obvious defects. It is noteworthy that a long-term inhibition of HSP90 will affect mitotic progression by impairing the centrosomal functions [23], [24], [25]. However, under this short period of inhibition condition, no obvious defect was detected. As expected, inhibition of proteasome delayed the progression to anaphase, since its activity is required for chromosome separation (**Figure 2C and D**).

### Celastrol disorganizes mitotic spindles

**Figure 3:**
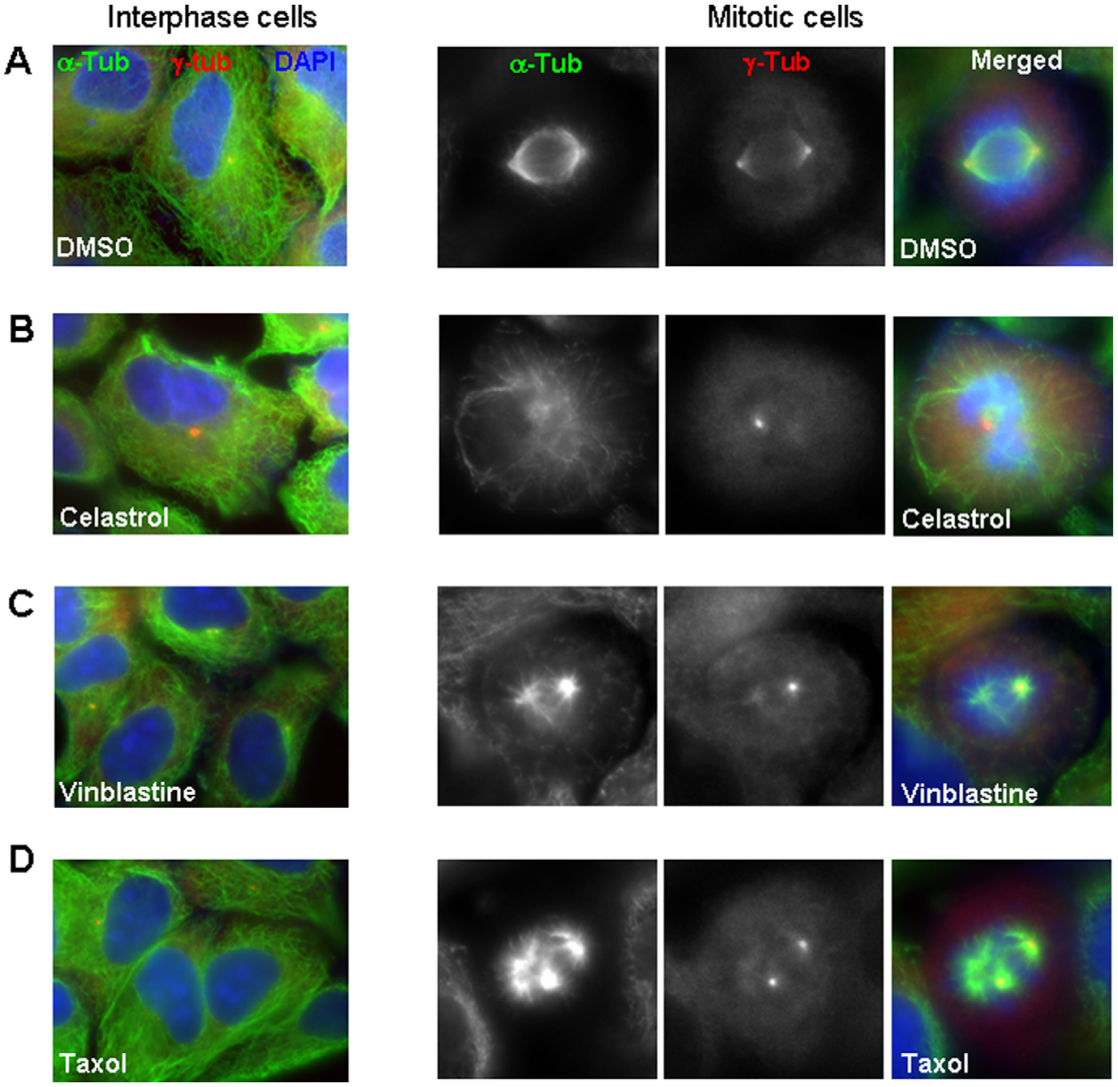
Celastrol disorganizes mitotic spindles. The representative immunostaining of microtubule (alpha- tubulin) and centrosome (gamma-tubulin) of the interphase and spindle morphology of mitotic HeLa cells treated for 1 hour with the indicated drugs; Celastrol (4 µM), Vinblastine (10 nM), and Taxol (10 nM).

To better understand the effects of Celastrol at the cellular level, we examined if the structure of MT was affected in treated cells. Surprisingly, at the concentration of Celastrol (2–4 µM) that inhibited cell migration and chromosome alignment, we found a relatively intact MT structure in interphase cells (**Figure 3A and B**). However, a significant disorganization of mitotic spindle was seen in mitotic cells (**Figure 3** **A and B**). During the prometaphase, the mitotic spindles and the associated motor proteins help align chromosomes at the metaphase plane. At this stage, apart from the centrosomal localization, gamma-tubulin is also localized to the spindles to help their assembly [1]. In epithelial cells, the mitotic spindle is assembled in parallel to substratum [26]. Consistent with this report, in most of control HeLa cells (95%, n = 25), two centrosomes as visualized by gamma-tubulin staining were observed in the same focal plane of 100 x objective lens (**Figure 3A** **and Figure S1A**). However, in Celastrol-treated cells, the spindle localization of gamma-tubulin was significantly abolished (93%, n = 42/45) and the plane of two poles with respect to substratum was severely distorted, leaving two centrosomes hardly in the same focal plane (78%, n = 35/45) or positioning them adjacent to each other (22%, n = 10/45) (**Figure 3B** **and Figure S1B**). These defects appeared to be different from those caused by Vinblastine, a MT depolymerizer, or Taxol a MT stabilizer, which mainly affect polymerization/depolymerization of MT (**Figure 3C and D**). This observation suggests that Celastrol disorganizes the mitotic spindles using a different mechanism.

### Celastrol causes structural defects of tubulin *in vitro* and reduces its level *in vivo*

**Figure 4:**
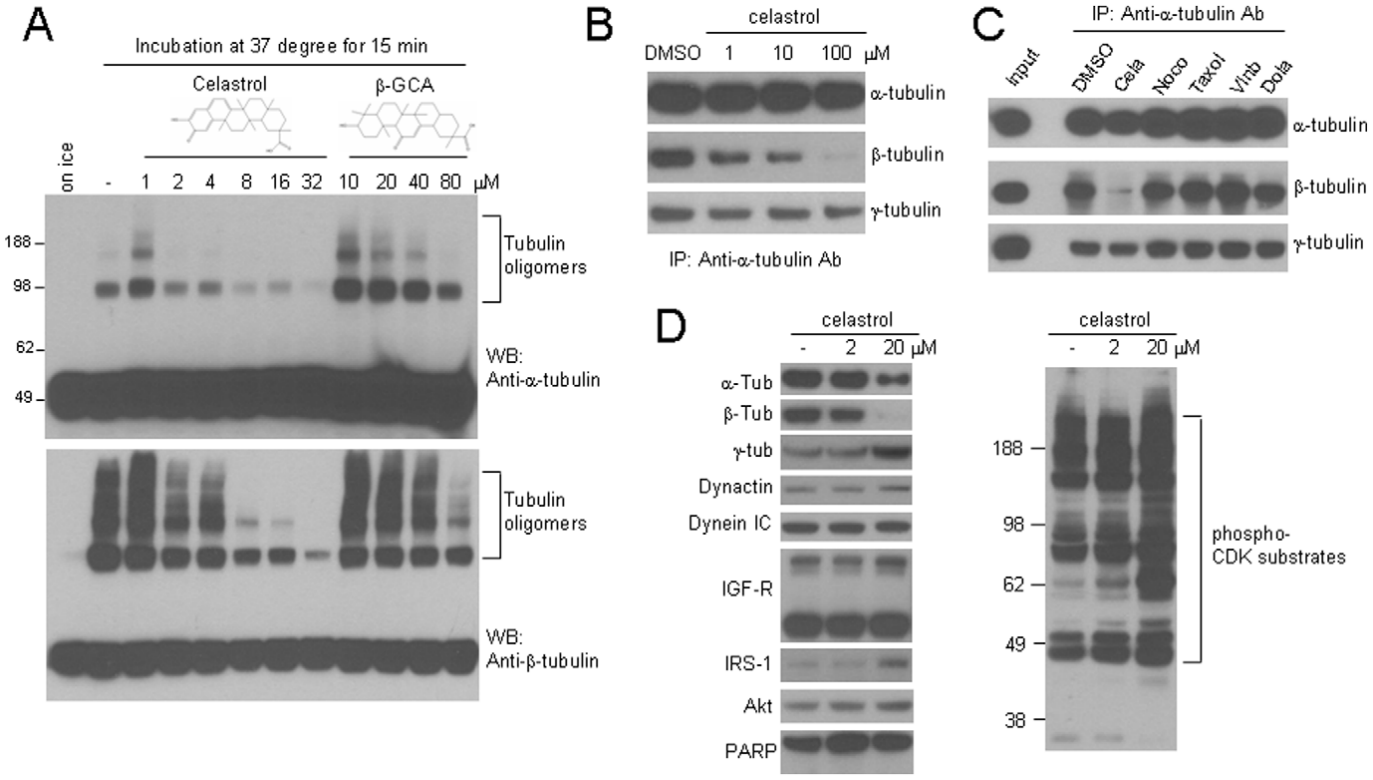
Celastrol causes structural defects of tubulin *in vitro* and reduces its level *in vivo*. (**A**) HEK293 cell lysates were incubated in the presence of the indicated amount of Celastrol or 18-beta GCA for 15 minutes prior to SDS-PAGE in non-reducing condition. The membrane was blotted with the alpha-tubulin (top) or beta-tubulin antibody (bottom). (**B**) The cell lysates from control HEK293 cells were prepared and equally divided into four different tubes, and immunoprecipitation was carried out with a polyclonal alpha-tubulin antibody in the presence of DMSO or indicated amounts of Celastrol. After SDS-PAGE, the immune complex was blotted with the mouse monoclonal antibody against the alpha-, beta-, or gamma-tubuln. The amount of alpha-tubulin (top panel) serves as a loading control. (**C**) The immunoprecipitation and Western blot was carried out as in B) in the presence of different chemicals. (**D**) HEK293 cells were treated with indicated amounts of Celastrol for 1 hour, and the whole cell lysates were analyzed for the indicated cellular proteins. The same lysates were blotted with the phospho-CDK substrate antibody (right panel).

The Celastrol-induced functional defects of MTs could be caused by inhibition of many cellular factors. We tested a possibility that tubulin itself could be a direct target. Apart from its well-established polymerization *in vitro*, which requires a high concentration (above 1–2 mg/ml) and GTP, the tubulin heterodimer manifests a variety of other biochemical properties, including the GTP-independent oligomerization, intra- and inter-molecular disulfide bond, non-disulfide bond cross-linking, and non-enzymatic cleavage [27], [28], [29], [30], [31]. We examined if Celstrol could affect any of these biochemical properties. As previously reported, incubation of purified tubulin led to oligomerization (or high molecular weight complexes) detectable on a non-reducing condition (**Figure 4A**). The majority but not all of these oligomers disappeared in a reducing condition (**Figure S2A**). The amounts of remaining tubulin oligomers were variable in each experiment and may represent SDS-resistant and non-disulfide cross-linked tubulins [31]. The addition of GTP, which stabilizes the tubulin structure, but not ATP, increased the level of oligomers in both HEK293 cell lysates and purified tubulins, indicating this oligomerization may require some degree of structural stability (**Figure S2B**). Using this assay with HEK293 cell lysate, we found that Celastrol inhibited formation of tubulin oligomers, whereas 18 beta-glycyrrhetic acid (18-βGCA), a triterpene structurally similar to Celastrol, showed a much weaker inhibitory effect (**Figure 4A**). A similar inhibitory effect was also observed when purified tubulin was used (**Figure S3**).

This inhibitory effect could be due to destabilization (i.e. unfolding) of tubulins in the presence of Celastrol. If this is the case, then Celastrol may disrupt the dimeric interaction between alph- and beta-tubulin. To directly test this possibility, we first prepared cell lysates from control HEK293 cells, and the alpha-tubulin was immunoprecipitated with a polyclonal antibody in the presence of different amounts of Celastrol. The level of beta- and gamma- tubulin in the resulting immunecomplex was examined. Intriguingly, the amount of beta-tubulin was decreased in a Celastrol-dose dependent manner, while that of gamma-tubulin remained largely unaffected (**Figure 4B**). When tested under the same experimental condition, the other MT drugs showed no such effect (**Figure 4C**), indicating that Celastrol may affect the structural integrity of tubulin heterodimer.

The biogenesis of tubulin heterodimer is tightly regulated, and the damaged tubulins are subjected to degradation or recycling pathway that involves the tubulin-specific cofactors [32]. To test if Celastrol affects the level of tubulin *in vivo*, we treated HEK293 cells with Celastrol for 1 hr, and the levels of tubulin along with other cellular proteins were examined. Interestingly, the level of both alpha- and beta-tubulin was significantly reduced, while that of MT-associated motor protein components (Dynactin p150 and dynein IC) or other known targets of the proteasome and HSP90 pathway, such as IGF receptor, IRS-1, and Akt was not changed (**Figure 4D**). The level of gamma-tubulin was slightly increased or unaffected by Celatrol treatment (**Figure 4D** **and Figure S4**). Perturbation of MT structures affects the activity of cyclin-dependent kinases (CDKs) *in vivo*, leading to an enhanced phosphorylation of target proteins [33], [34]. The reduction of tubulin level by Celastrol may lead to MT structural defects and affect the phosphorylation status of CDKs target proteins. Consistent with this prediction, the levels of phosphorylation of CDK-target proteins were greatly increased in the presence of Celastrol (**Figure 4D**). Collectively, these results suggest that Celastrol alters MT functions by directly targeting tubulin heterodimer.

### Taxol-resistant cancer cells remain sensitive to Celastrol-induced apoptosis

**Figure 5:**
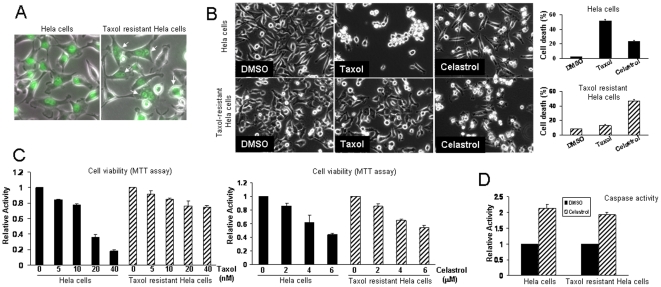
Taxol-resistant cancer cells remain sensitive to Celastrol-induced apoptosis. (**A**) The multinuclei phenotype of Taxol-resistant HeLa cells expressing Histone H2B-EGFP. Arrows indicate the cells with the multiple nuclei, and approximately 50% of cells displayed this phenotype. (**B**) The bright field images of parental and Taxol-resistant cells treated with Taxol (20 nM) for 36 hour or Celastrol (4 µM) for 24 hours. The percentage of dead cells was quantified based on the nuclear morphology as determined by Histone H2B-EGFP, and cells with the fragmented nuclei were counted as dead cells (right panel). (**C**) The sensitivity of parental and Taxol-resistant cells toward Taxol or Celastrol. Each cell line was treated with the indicated amounts of Taxol for 36 hours or Celastrol for 24 hours, and the cell viability was determined by MTT assay. The relative activity with respect to vehicle alone (DMSO) was presented. (**D**) The parental and Taxol-resistant cells were treated with DMSO or Celastrol (4 µM) for 6 hours, and the cytosolic cell lysates were subjected to caspase activity assay. The relative caspase activity over DMSO treatment was presented.

The MT stabilizer Taxol is one of the most widely used anti-cancer agents. However, some cancer cells develop a resistance, and such resistant cancer cells may further accumulate the genomic instability, contributing to more malignant transformation. Celastrol has been shown to have anti-cancer properties [19], [20], [21], [35]. We demonstrated that it regulates MT function via a different mechanism. Thus we next explored if Celastrol could be still effective to Taxol-resistant cancer cells. For this purpose, we generated a Taxol-resistant HeLa cell line, which constitutively express EGFP-H2B proteins, allowing us to identify cells at each cell-cycle stage. In any given time, approximately 50% of Taxol-resistant cells displayed the multiple nuclei phenotype, indicating the uncoupling of chromosome segregation with cytokinesis (**Figure 5A**). Compared with the parental cells, Taxol-resistant cells showed a significant resistance toward Taxol-induced cell death (**Figure 5B**). However, upon Celastrol treatment, these cells still manifested a similar dose-dependent reduction of cell viability as the parental cells (**Figure 5C**). These results were further confirmed by a similar dose-dependent sensitivity and apoptotic capacity of Taxol-resistant cells when treated with Celastrol, compared to normal Hela cells (**Figure 5D**).

### Celastrol selectively kills mitotic arrested cells

Regardless of differences in the mode of action, the utility of MT drugs as anti-cancer agents is primarily due to their ability to cause mitotic cell death. However, inhibition of MT functions can also lead to perturbation of normal cellular functions. Therefore, chemicals that facilitate mitotic death (i.e. proliferating cancer cells) present a great potential to enhance the efficacy of MT drugs. We found that Celastrol showed a synergistic apoptotic activity when sequentially treated with various conventional MT drugs (**Figure S5**). However, it is not known if Celastrol preferentially affect the mitotic-arrested cells or drug-exposed interphase cells. To specifically address this issue, we employed a time-lapse live cell imaging and tracking assay using Hela cells expressing EGFP-H2B protein. The drug-arrested mitotic cells are easily distinguishable from the drug-exposed interphase cells, based on the GFP-positive chromosome structure and their round shape morphology (**Figure S6A**). Also, the apoptotic cells can be identified when the plasma membrane collapses (or ‘membrane blebbing’) as they die (**Figure S6B and C**).

**Figure 6:**
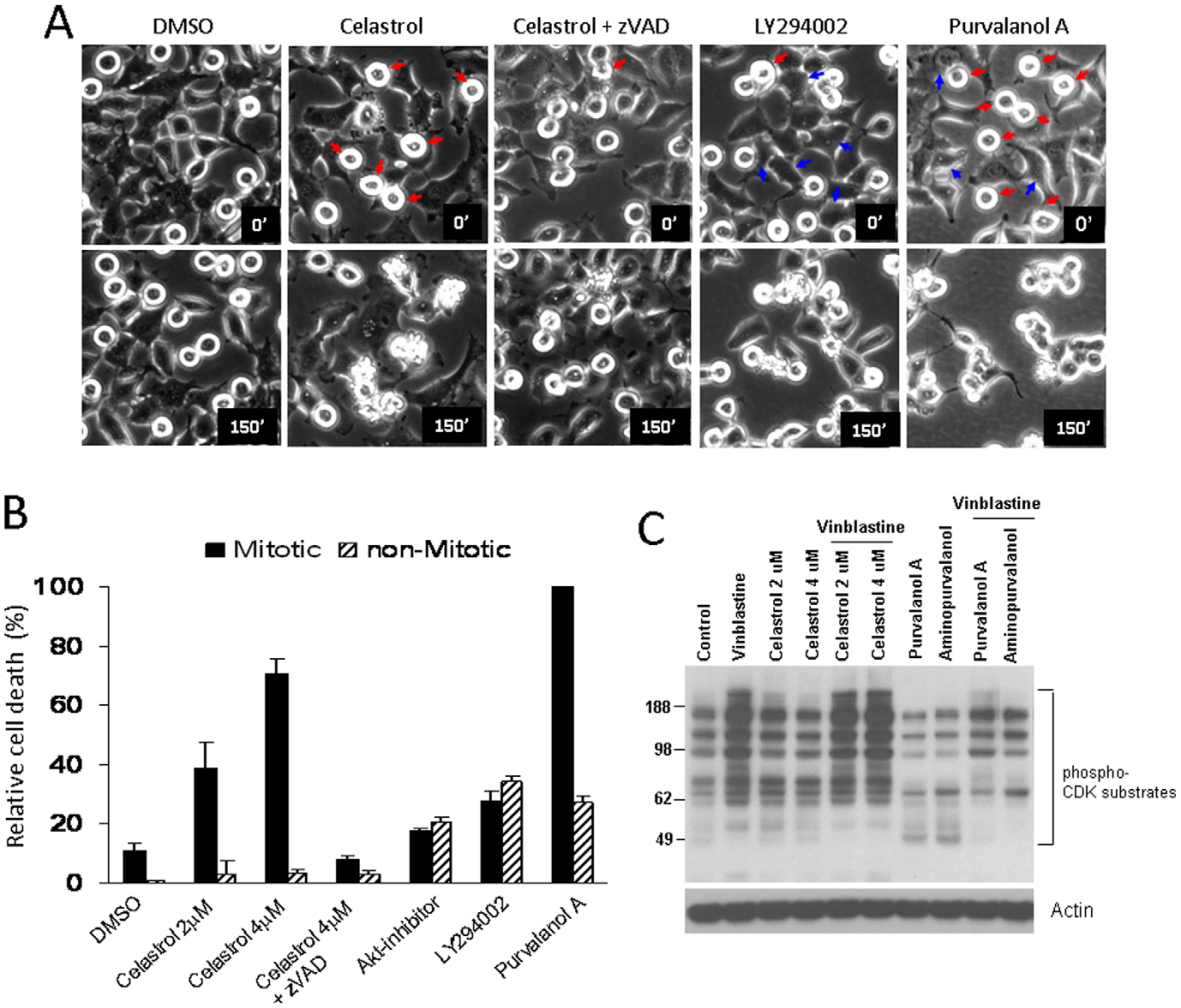
Celastrol selectively kills mitotic arrested cells. (**A**) The time-lapse images of Vinblastine-arrested cells treated with different chemicals. The magnified still images were shown for the clarity. The pre-arrested mitotic or interphase cells to die within 150 minutes were indicated by red and blue arrows, respectively. (**B**) Quantification of mitotic or non-mitotic cell death. The percentage of dying cells over total number of cells in mitotic or interphase state at time 0 was presented. (**C**) Celastrol did not inhibit Vinblastine-induced phosphorylation of CDK target proteins. Cells were treated with each drug for 1 hour or pre-treated with Vinblastine (100 nM) for 30 minutes followed by subsequent drug treatment for 1 hour. The whole cell lysate was analyzed with the phospho-CDKs substrate antibody, and the same blot was probed with the actin antibody for loading control.

Cells were pre-treated with Vinblastine (10 nM) for 4 hours to induce mitotic arrest. Then, various pharmacological agents were subsequently added and the time-lapse images were taken for 4 hours. The apoptotic death of pre-arrested mitotic cells and non-mitotic cells were assessed (**Figure 6A and B**). Within this experimental time period, Vinblastine alone failed to induce cell death, although it induced mitotic arrest. This was also the case even in the presence of a higher concentration (100 nM) of Vinblastine for up to 12 hours (data not shown).

Intriguingly, when subsequently treated with Celastrol, the pre-arrested mitotic cells preferentially underwent apoptosis (**Table 1** **,** **Figure 6A and B**). Cell death was reversed by co-treatment of a caspase inhibitor zVAD, suggesting caspase-mediated apoptotic pathway is involved in this process (**Table 1** **,** **Figure 6A and B**). The inhibitors of other cellular pathways, such as HSP90 and NF-κB, known to be affected by Celastrol, had no such effect. Inhibition of PI3K-Akt pathway, a well-known cell survival signal, failed to show any noticeable selective effects. In contrast, Akt inhibitor and LY294002 (a PI3K inhibitor) appeared to enhance apoptosis of both mitotic and non-mitotic cells (**Figure 6A and B**). To determine the effects of Celastrol alone in control mitotic cells, we first synchronized cells at G1/S phase by double-thymidine block method [17] and the fate of cells entering mitosis was examined (see Materials and Methods). Although the efficiency was much reduced, compared with the ‘Vinblastine-arrest’ mitotic cells, a significant fraction of normal mitotic cell also underwent apoptosis (**Table 1**), suggesting that mitotic cells are intrinsically sensitive to Celastrol-induced cell death. This finding may explain the synergistic apoptotic activity of Celastrol in combination with various microtubule drugs, including Taxol, which induce mitotic arrest by stabilizing the microtubules (Figure S5). Previously, it has been shown that genetic or pharmacological inhibition of CDKs led to a massive cell death in Taxol-arrested cancer cells [33]. Consistent with this finding, we also observed an increased apoptosis in the presence of CDK inhibitors (**Table 1** **and Figure S7**). However, unlike the inhibitors of CDKs, Celastrol did not inhibit either the basal or Vinblastine-induced phosphorylation of CDK target proteins (**Figure 6C**). In addition, in contrast to Celastrol, we found no obvious MT defects in both interphase and mitotic cells treated with CDK inhibitors (**Figure S8**). These findings indicate that the molecular mechanisms by which Celastrol enhances the mitotic death are different or, at least, not through direct inhibition of CDK activity.

**Table 1:** Effect of pharmacological agents on mitotic cell death.

Tested chemicals	Targets	# cells examined	# cells dead	% cell death
Vinblastine Only	MT destablizer	98	11	11.2
Celastrol only (4 µM)	Tubulin binding	72	25	34.7
Celastrol (2 µM)		69	27	39.1
Celastrol (4 µM)		61	43	70.5
18-bGCA (20 µM)	Structurally analogous	22	4	18.2
	to Celastrol			
17AAG (8 µM)	HSP90	16	2	12.5
MG132 (5 µM)	Proteasome	30	0	0
Etoposide (10 µM)	Topoisomerase	30	6	20
Bay 11-7085 (20 µM)	NF-kappa B	27	1	3.7
Resveratrol (20 µM)	Antioxidant	22	1	4.5
Akt-i VIII (7.6 µM)	Akt	28	5	17.9
LY294002 (20 µM)	PI3K	36	10	27.8

HeLa cells expressing EGFP-Histone H2B (HeLa-H2B) were arrested at mitotic phase by Vinblastine treatment, and the indicated amounts of each drug was added. The apoptotic death of only the ‘pre-arrested’ mitotic cells in 4 hours as identified by time-lapse movie was scored. ‘Vinblastine only’ indicates without addition of other drugs and ‘Celastrol only’ indicates its effect on normal mitotic cells (see Materials and Methods).

## Discussion

Chemical compounds from natural sources continue to serve as important pharmacological tools for the understanding of biological pathways and provide critical chemical platforms for new drug discovery. The utility of various anti-microtubule agents from natural origins is the best example that continuously serves these two purposes. In this study, we identified a novel tubulin-targeting activity of a natural product Celastrol, and demonstrated that this unique activity, in combination with conventional microtubule-targeting anti-cancer drugs, could be exploited to selectively induce apoptotic cell death of mitotic cells.

Identification of tubulin as a primary target of Celastrol explains many of its cellular and biological effects. For instance, Celastrol was derived from the plant extracts used for the treatment of inflammatory symptoms in traditional medicine [18]. This anti-inflammatory effect can be directly attributed to inhibition of MT-mediated cellular processes, such as migration of immune cells and secretion of inflammatory cytokines. This view is consistent with the fact that Colchicine, an MT destabilizer, is one of the earliest medicines used for the treatment of inflammatory gout disease. This initial effect could be accompanied by the subsequent transcriptional repression of pro-inflammatory genes through inhibition of NF-κB activation by Celastrol [22], [36].

Some of the anticancer activity of Celastrol may come from inhibition of proteasome, HSP90, or NF-κB pathway as previously reported [19], [20], [21], [22], [36]. However, the known inhibitors of these pathways had little effect on apoptosis of MT drug-arrested mitotic cells. Therefore, under this experimental condition, inhibition of these pathways does not appear to be the main cause of Celastrol-induced mitotic cell death. Intriguingly, in a gene expression-based *in silico* screen, Celastrol was identified as a compound with the ‘parthenolide (PTL)-like’ gene expression signature. It was shown that, like PTL, Celastrol selectively eradicated leukemic stem cells through inhibition of NF-κB survival pathway [35]. However, of note is the report that PTL inhibited the antigen-induced microtubule formation and degranulation of master cells independent of NF-κB inhibition [37]. This finding is further supported by a recent discovery of PTL as a chemical inhibitor of tubulin carboxypeptidase, which involves in the regulation of alpha tubulin tyrosination [38]. Thus, it appears that, like Celastrol, the microtubule-inferring activity of PTL can also contribute to its anti-cancer properties.

The molecular mechanisms underlying the enhanced killing of MT drug-arrested mitotic cells by Celastrol are the subject of future investigations. However, we ruled out the possibility that Celastrol simply inhibited the cell survival pathway(s). For example, it may be speculated that, due to a loss or reduction of the contact with the substratum, the MT drug-arrested mitotic cells are intrinsically more susceptible to apoptotic stimuli. If this is the case, inhibition of general cell survival pathways, such as PI3K-Akt and NF-κB, may result in a similar selective killing effect. However, our data did not support this prediction. Other inhibitors of the cellular pathways, with which Celastrol was reported to interfere, also failed to exert a similar effect. Another possibility might be that Celastrol inhibits the activity of CDKs, which have been shown to result in a massive cell death in Taxol-arrested cancer cells [33]. Consistent with this finding, the chemical inhibitors of CDKs led to apoptosis in a manner correlated with their activity to inhibit the phosphorylation of target proteins. However, Celastrol did not affect the phosphorylation status of CDKs target proteins, making this possibility also very unlikely.

What could be responsible for this selective killing effect? We showed that Vinblastine alone was not sufficient to induce mitotic cell death within our experimental time period, whereas Celastrol alone caused some degree of apoptosis, indicating that mitotic cells are intrinsically sensitive to Celastrol. This observation is consistent with the synergistic apoptotic effect of Celastrol with various MT drugs that induce mitotic arrest in different mechanisms. We showed that, unlike other MT drugs, Celastrol disrupted tubulin heterodimer *in vitro*. Can disrupting tubulin heterodimer be a contributing factor for cell death? This possibility assumes that the structural defects or modifications of tubulin perturb the additional cellular pathways that contribute to cell death. This view is consistent with the studies showing that the cytotoxic effects of natural and synthetic chemicals are closely associated with their activity to target tubulins [39], [40], [41], [42], [43]. Celastrol may also cause an enhanced turnover of tubulin heterodimer through the structural defects. We showed that Celastrol directly affected the structural integrity of tubulin *in vitro*. It could be possible that such binding may influence the *in vivo* modifications of tubulin, such as tyrosination. In this respect, it is important to point out the identification of Celastrol as a chemical with the ‘PTL-like’ functional property [35]. PTL has anti-cancer activities, and was shown to inhibit tubulin tyrosination [44], which is required for the recruitment of the CAP-Gly (cytoskeleton-associated protein-glycine-rich) domain-containing proteins to MTs. Defects in tubulin tyrosination led to mislocalization of these proteins, resulting in spindle defects during mitosis [6], [44]. The mitotic spindle defects caused by Celastrol could be attributable to such changes.

In summary, we identified a novel tubulin-targeting activity of Celastrol and demonstrated its utility as an enhancer of mitotic cell death. Therefore, we provide a molecular explanation for its anti-inflammatory and anti-cancer activity, and present a chemical tool to elucidate the molecular mechanisms underlying the mitotic cell death and to enhance the efficacy of MT drugs.

## Supporting Information

Figure S1 Celastrol causes defects in centrosomal positioning in mitotic cells. The representative staining of gamma- and alpha-tubulin in mitotic cells treated with DMSO (A) or Celastrol (4 uM) for 1 hour (B). The prometaphase or metaphase of control mitotic cells was identified based on chromosomal morphology as visualized by DAPI staining. The images of Celastrol-treated mitotic cells were taken at two different focal planes, focusing on each gamma-tubulin, and the corresponding alpha-tubulin staining was shown to reveal the degree of spindle disorganization.
(0.69 MB TIF)

Figure S2 Oigomerization of purified tubulin. A) The purified tubulin was incubated for the indicated time and run on SDS-PAGE in non-reducing or reducing condition, and blotted with the alpha-tubulin antibody. B) HEK293 cell lysates or purified tubulin was incubated in the presence or absence of ATP or GTP for 15 minutes, and analyzed for the oligomer formation in non-reducing condition.
(0.14 MB TIF)

Figure S3 Celastrol inhibits oligomerization of purified tubulin. The purified tubulin was incubated in the presence of increasing amount of Celastrol or 18-beta GCA for 60 minutes, and analyzed with the mouse monoclonal alpha-tubulin antibody.
(0.11 MB TIF)

Figure S4 The level of gamma-tubulin was unaffected by Celatrol treatment. HEK293 cells were treated with the different amounts of Celastrol for 1 hour and the whole cell lysates were analyzed for the level of beta-tubulin, gamma- tubulin, or Akt.
(0.08 MB TIF)

Figure S5 The synergistic apoptotic effects of Celastrol with conventional microtubule drugs. Cells were pre-treated with each microtubule drug (10 nM) for 4 hour and then treated with Celastrol (4 uM) for additional 4 hour prior to the caspase activity assay. The relative activity was normalized against Celastrol treatment (4 hr) only.
(0.06 MB TIF)

Figure S6 Identification of mitotic cells and apoptotic cells. A) The cell shape and chromosomal morphology of the interphase or mitotic-arrested cells. B) Apoptotic death of mitotic cells. Numbers indicate time after Vinblastine (100 nM) treatment. C) Apoptotic death of non-mitotic cells pre-treated with Vinblastine (10 nM for 4 hours) followed by LY294002 treatment (20 uM). Numbers indicate time after LY294002 (see Figure 6 for details).
(0.32 MB TIF)

Figure S7 Effects of chemical inhibitors of CDKs on mitotic cell death. Cells were pre-treated with Vinblastine (10 nM) for 4 hours and subsequently treated with each chemical (10 uM) for additional 4 hours.
(0.26 MB TIF)

Figure S8 CDK inhibitor does not cause microtubule defects. Representative immunostaining of interphase microtubules and mitotic spindles of HeLa cells treated with Purvalanol A (10 uM) for 1 hour.
(0.39 MB TIF)

Movie S1 fMLP-induced neutrophil chemotaxis
(0.72 MB WMV)

Movie S2 Effect of Celastrol on neutrophil chemotaxis
(0.73 MB WMV)

Movie S3 Migration of Hela cells toward wounded area.
(0.64 MB AVI)

Movie S4 Effect of Celastrol on migration of Hela cells.
(0.64 MB AVI)

Movie S5 Chromosomal behavior of mitotic cells.
(1.15 MB AVI)

Movie S6 Effect of Celastrol on behavior of mitotic chromosomes.
(1.15 MB AVI)

## References

[1] Luders J, Stearns T (2007). Microtubule-organizing centres: a re-evaluation.. Nat Rev Mol Cell Biol.

[2] Hammond JW, Cai D, Verhey KJ (2008). Tubulin modifications and their cellular functions.. Curr Opin Cell Biol.

[3] Janke C, Rogowski K, Wloga D, Regnard C, Kajava AV (2005). Tubulin polyglutamylase enzymes are members of the TTL domain protein family.. Science.

[4] Gaertig J, Wloga D (2008). Ciliary tubulin and its post-translational modifications.. Curr Top Dev Biol.

[5] Weisbrich A, Honnappa S, Jaussi R, Okhrimenko O, Frey D (2007). Structure-function relationship of CAP-Gly domains.. Nat Struct Mol Biol.

[6] Steinmetz MO, Akhmanova A (2008). Capturing protein tails by CAP-Gly domains.. Trends Biochem Sci.

[7] Fukasawa K (2007). Oncogenes and tumour suppressors take on centrosomes.. Nat Rev Cancer.

[8] Hunt JT (2009). Discovery of ixabepilone.. Mol Cancer Ther.

[9] Mooberry SL (2007). Strategies for the development of novel Taxol-like agents.. Methods Mol Med.

[10] Burris HA (2008). Preclinical investigations with epothilones in breast cancer models.. Semin Oncol.

[11] Nagle A, Hur W, Gray NS (2006). Antimitotic agents of natural origin.. Curr Drug Targets.

[12] Shi J, Orth JD, Mitchison T (2008). Cell type variation in responses to antimitotic drugs that target microtubules and kinesin-5.. Cancer Res.

[13] Symmans WF, Volm MD, Shapiro RL, Perkins AB, Kim AY (2000). Paclitaxel-induced apoptosis and mitotic arrest assessed by serial fine-needle aspiration: implications for early prediction of breast cancer response to neoadjuvant treatment.. Clin Cancer Res.

[14] Milross CG, Mason KA, Hunter NR, Chung WK, Peters LJ (1996). Relationship of mitotic arrest and apoptosis to antitumor effect of paclitaxel.. J Natl Cancer Inst.

[15] Zhu D, Hattori H, Jo H, Jia Y, Subramanian KK (2006). Deactivation of phosphatidylinositol 3,4,5-trisphosphate/Akt signaling mediates neutrophil spontaneous death.. Proc Natl Acad Sci U S A.

[16] Jo H, Jia Y, Subramanian KK, Hattori H, Luo HR (2008). Cancer cell-derived clusterin modulates the phosphatidylinositol 3′-kinase-Akt pathway through attenuation of insulin-like growth factor 1 during serum deprivation.. Mol Cell Biol.

[17] Whitfield ML, Zheng LX, Baldwin A, Ohta T, Hurt MM (2000). Stem-loop binding protein, the protein that binds the 3′ end of histone mRNA, is cell cycle regulated by both translational and posttranslational mechanisms.. Mol Cell Biol.

[18] Corson TW, Crews CM (2007). Molecular understanding and modern application of traditional medicines: triumphs and trials.. Cell.

[19] Yang H, Chen D, Cui QC, Yuan X, Dou QP (2006). Celastrol, a triterpene extracted from the Chinese “Thunder of God Vine,” is a potent proteasome inhibitor and suppresses human prostate cancer growth in nude mice.. Cancer Res.

[20] Hieronymus H, Lamb J, Ross KN, Peng XP, Clement C (2006). Gene expression signature-based chemical genomic prediction identifies a novel class of HSP90 pathway modulators.. Cancer Cell.

[21] Zhang T, Hamza A, Cao X, Wang B, Yu S (2008). A novel Hsp90 inhibitor to disrupt Hsp90/Cdc37 complex against pancreatic cancer cells.. Mol Cancer Ther.

[22] Sethi G, Ahn KS, Pandey MK, Aggarwal BB (2007). Celastrol, a novel triterpene, potentiates TNF-induced apoptosis and suppresses invasion of tumor cells by inhibiting NF-kappaB-regulated gene products and TAK1-mediated NF-kappaB activation.. Blood.

[23] de Carcer G (2004). Heat shock protein 90 regulates the metaphase-anaphase transition in a polo-like kinase-dependent manner.. Cancer Res.

[24] de Carcer G, do Carmo Avides M, Lallena MJ, Glover DM, Gonzalez C (2001). Requirement of Hsp90 for centrosomal function reflects its regulation of Polo kinase stability.. Embo J.

[25] Prosser SL, Straatman KR, Fry AM (2009). Molecular dissection of the centrosome overduplication pathway in S-phase-arrested cells.. Mol Cell Biol.

[26] Toyoshima F, Matsumura S, Morimoto H, Mitsushima M, Nishida E (2007). PtdIns(3,4,5)P3 regulates spindle orientation in adherent cells.. Dev Cell.

[27] Martin SR, Clark DC, Mayley PM (1982). Interactions of tubulin and microtubule-associated proteins. Conformation and stability of the oligomeric species from glycerol-cycled microtubule protein of bovine brain.. Biochem J.

[28] Correia JJ, Lipscomb LD, Dabrowiak JC, Isern N, Zubieta J (1994). Cleavage of tubulin by vanadate ion.. Arch Biochem Biophys.

[29] Carlier MF, Simon C, Pantaloni D (1984). Polymorphism of tubulin oligomers in the presence of microtubule-associated proteins. Implications in microtubule assembly.. Biochemistry.

[30] Barton JS, Riazi GH (1982). Evidence against tubulin oligomer dissociation to tubulin dimer at assembly temperatures.. Biochim Biophys Acta.

[31] Correia JJ, Lipscomb LD, Lobert S (1993). Nondisulfide crosslinking and chemical cleavage of tubulin subunits: pH and temperature dependence.. Arch Biochem Biophys.

[32] Lewis SA, Tian G, Cowan NJ (1997). The alpha- and beta-tubulin folding pathways.. Trends Cell Biol.

[33] O'Connor DS, Wall NR, Porter AC, Altieri DC (2002). A p34(cdc2) survival checkpoint in cancer.. Cancer Cell.

[34] Morgan DO (1997). Cyclin-dependent kinases: engines, clocks, and microprocessors.. Annu Rev Cell Dev Biol.

[35] Hassane DC, Guzman ML, Corbett C, Li X, Abboud R (2008). Discovery of agents that eradicate leukemia stem cells using an in silico screen of public gene expression data.. Blood.

[36] Jin HZ, Hwang BY, Kim HS, Lee JH, Kim YH (2002). Antiinflammatory constituents of Celastrus orbiculatus inhibit the NF-kappaB activation and NO production.. J Nat Prod.

[37] Miyata N, Gon Y, Nunomura S, Endo D, Yamashita K (2008). Inhibitory effects of parthenolide on antigen-induced microtubule formation and degranulation in mast cells.. Int Immunopharmacol.

[38] Fonrose X, Ausseil F, Soleilhac E, Masson V, David B (2007). Parthenolide inhibits tubulin carboxypeptidase activity.. Cancer Res.

[39] Mi L, Gan N, Cheema A, Dakshanamurthy S, Yang DC (2009). Cancer preventive isothiocyanates induce selective degradation of cellular alpha- and beta-tubulins by proteasomes.. J Biol Chem.

[40] Mi L, Xiao Z, Hood BL, Dakshanamurthy S, Wang X (2008). Covalent binding to tubulin by isothiocyanates. A mechanism of cell growth arrest and apoptosis.. J Biol Chem.

[41] Banerjee M, Poddar A, Mitra G, Surolia A, Owa T (2005). Sulfonamide drugs binding to the colchicine site of tubulin: thermodynamic analysis of the drug-tubulin interactions by isothermal titration calorimetry.. J Med Chem.

[42] Ross-Macdonald P, de Silva H, Guo Q, Xiao H, Hung CY (2008). Identification of a nonkinase target mediating cytotoxicity of novel kinase inhibitors.. Mol Cancer Ther.

[43] Lin YF, Tsai WP, Liu HG, Liang PH (2009). Intracellular beta-tubulin/chaperonin containing TCP1-beta complex serves as a novel chemotherapeutic target against drug-resistant tumors.. Cancer Res.

[44] Peris L, Thery M, Faure J, Saoudi Y, Lafanechere L (2006). Tubulin tyrosination is a major factor affecting the recruitment of CAP-Gly proteins at microtubule plus ends.. J Cell Biol.

