# Supplementary figures and images for: Natural Product Celastrol Destabilizes Tubulin Heterodimer and Facilitates Mitotic Cell Death Triggered by Microtubule-Targeting Anti-Cancer Drugs
Source: PLoS One. 2010 Apr 23;5(4):e10318. doi: 10.1371/journal.pone.0010318 (PMC2859055; doi:10.1371/journal.pone.0010318)

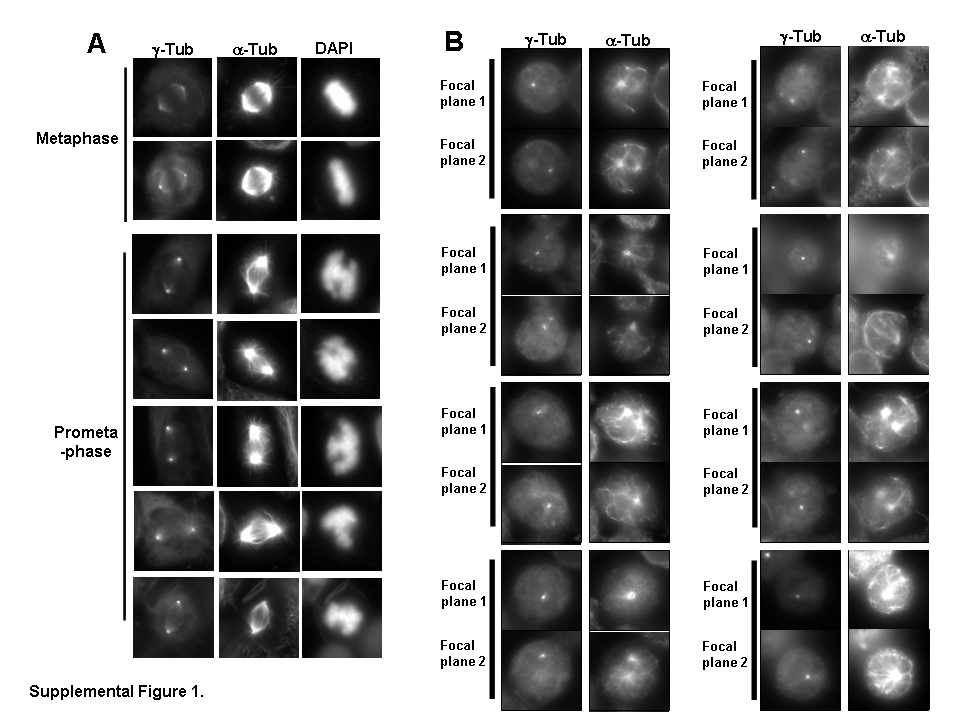

Supplement: Figure S1 — Celastrol causes defects in centrosomal positioning in mitotic cells. The representative staining of gamma- and alpha-tubulin in mitotic cells treated with DMSO (A) or Celastrol (4 uM) for 1 hour (B). The prometaphase or metaphase of control mitotic cells was identified based on chromosomal morphology as visualized by DAPI staining. The images of Celastrol-treated mitotic cells were taken at two different focal planes, focusing on each gamma-tubulin, and the corresponding alpha-tubulin staining was shown to reveal the degree of spindle disorganization. (0.69 MB TIF) [file pone.0010318.s001.tif]

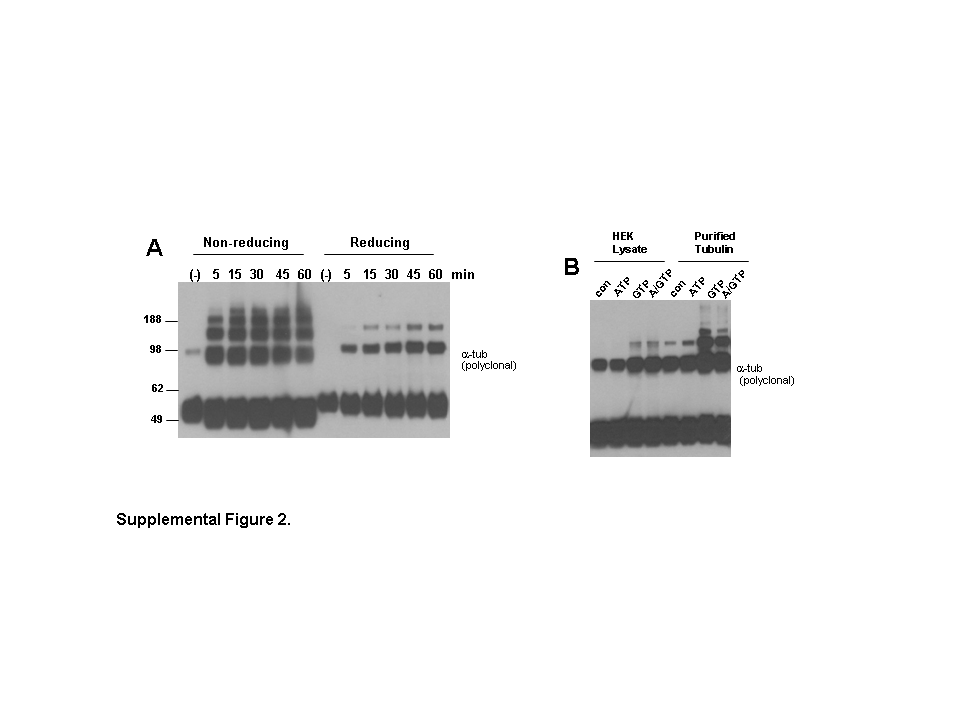

Supplement: Figure S2 — Oigomerization of purified tubulin. A) The purified tubulin was incubated for the indicated time and run on SDS-PAGE in non-reducing or reducing condition, and blotted with the alpha-tubulin antibody. B) HEK293 cell lysates or purified tubulin was incubated in the presence or absence of ATP or GTP for 15 minutes, and analyzed for the oligomer formation in non-reducing condition. (0.14 MB TIF) [file pone.0010318.s002.tif]

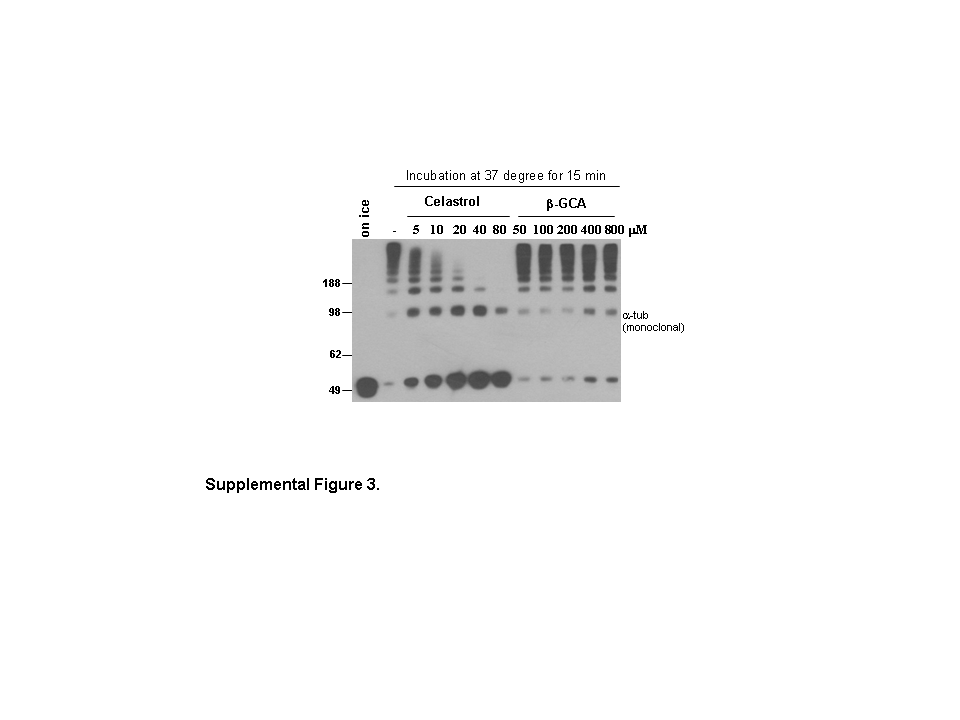

Supplement: Figure S3 — Celastrol inhibits oligomerization of purified tubulin. The purified tubulin was incubated in the presence of increasing amount of Celastrol or 18-beta GCA for 60 minutes, and analyzed with the mouse monoclonal alpha-tubulin antibody. (0.11 MB TIF) [file pone.0010318.s003.tif]

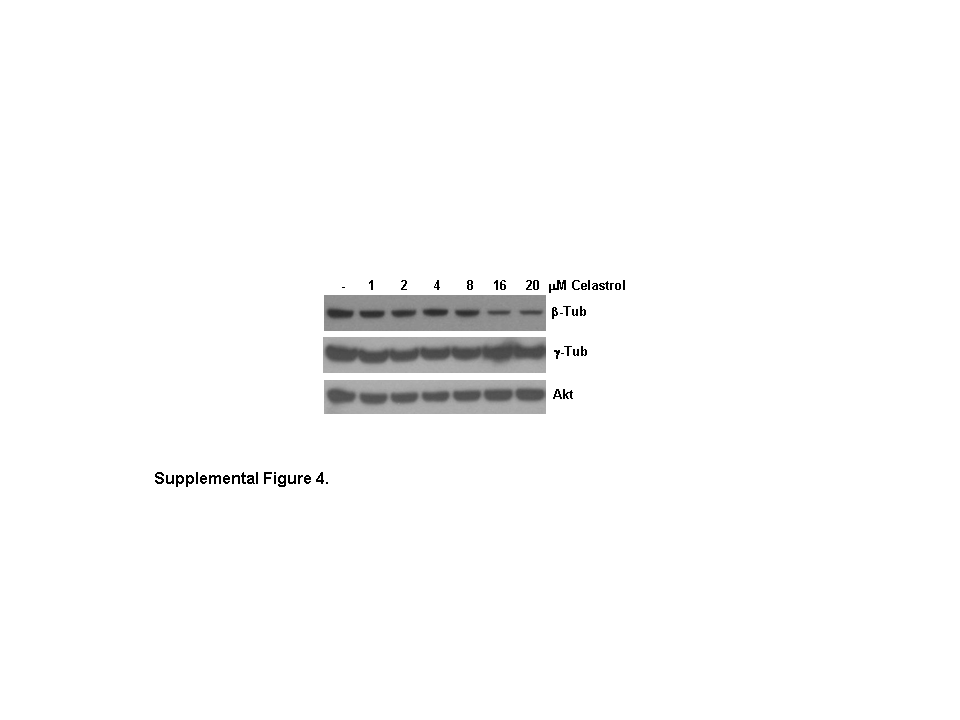

Supplement: Figure S4 — The level of gamma-tubulin was unaffected by Celatrol treatment. HEK293 cells were treated with the different amounts of Celastrol for 1 hour and the whole cell lysates were analyzed for the level of beta-tubulin, gamma- tubulin, or Akt. (0.08 MB TIF) [file pone.0010318.s004.tif]

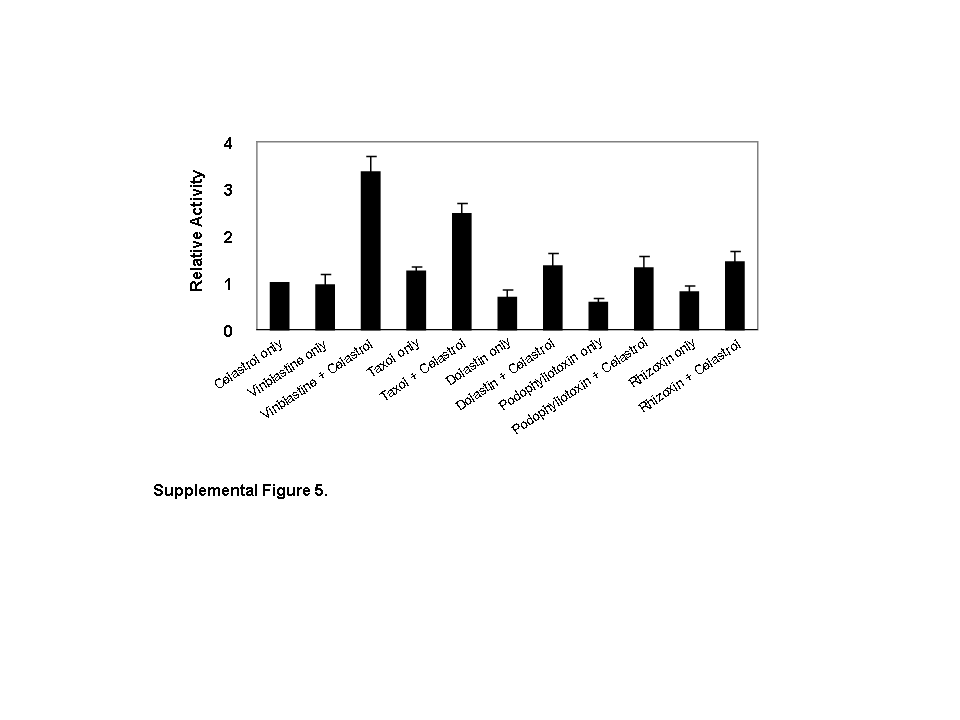

Supplement: Figure S5 — The synergistic apoptotic effects of Celastrol with conventional microtubule drugs. Cells were pre-treated with each microtubule drug (10 nM) for 4 hour and then treated with Celastrol (4 uM) for additional 4 hour prior to the caspase activity assay. The relative activity was normalized against Celastrol treatment (4 hr) only. (0.06 MB TIF) [file pone.0010318.s005.tif]

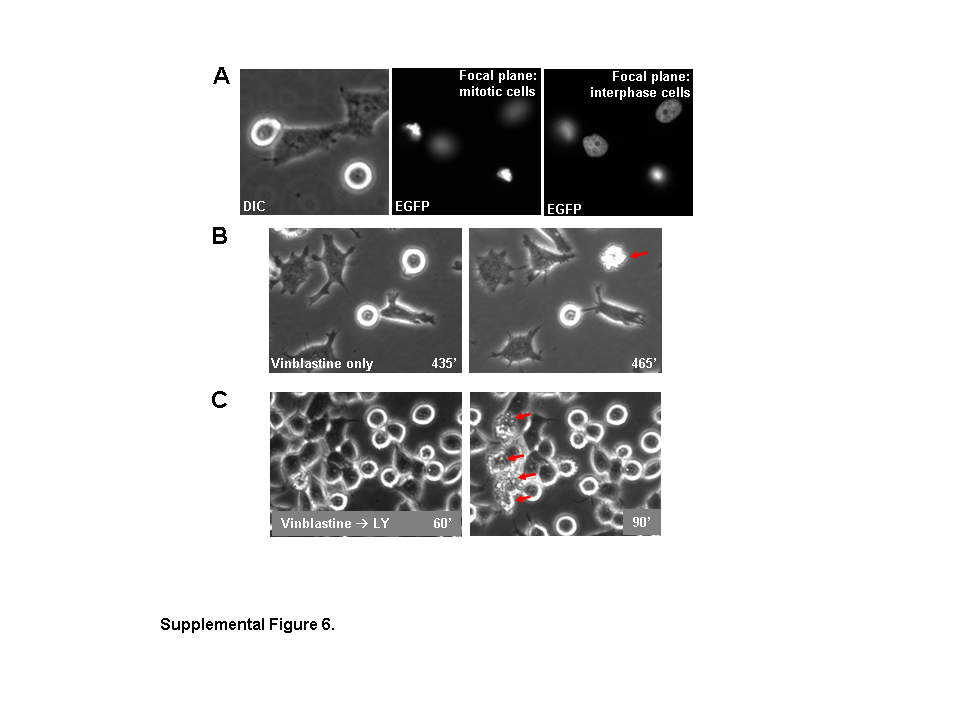

Supplement: Figure S6 — Identification of mitotic cells and apoptotic cells. A) The cell shape and chromosomal morphology of the interphase or mitotic-arrested cells. B) Apoptotic death of mitotic cells. Numbers indicate time after Vinblastine (100 nM) treatment. C) Apoptotic death of non-mitotic cells pre-treated with Vinblastine (10 nM for 4 hours) followed by LY294002 treatment (20 uM). Numbers indicate time after LY294002 (see Figure 6 for details). (0.32 MB TIF) [file pone.0010318.s006.tif]

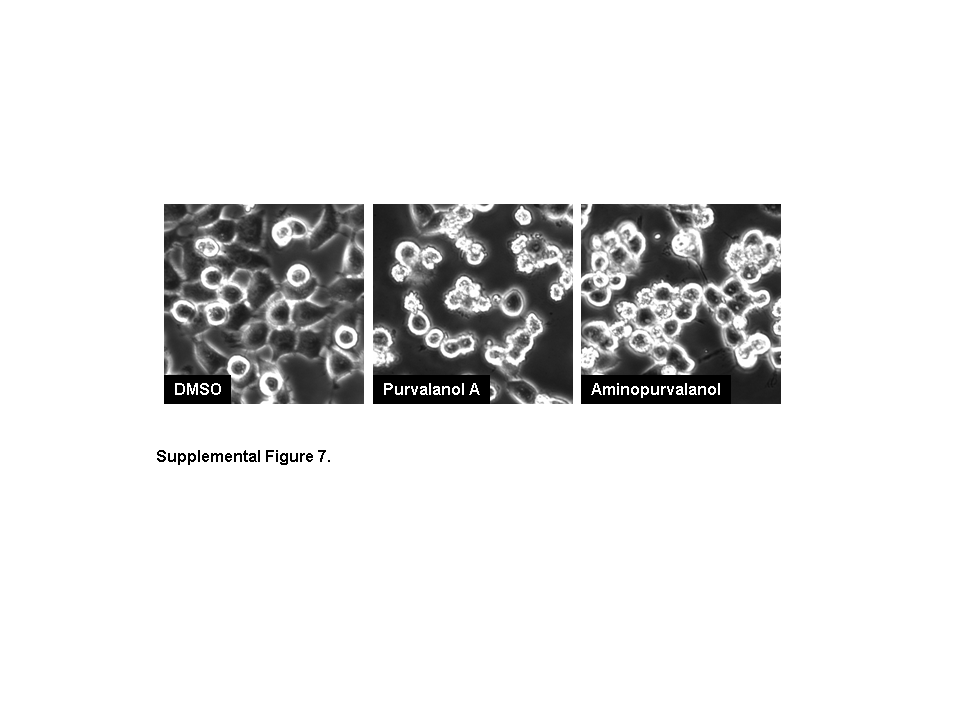

Supplement: Figure S7 — Effects of chemical inhibitors of CDKs on mitotic cell death. Cells were pre-treated with Vinblastine (10 nM) for 4 hours and subsequently treated with each chemical (10 uM) for additional 4 hours. (0.26 MB TIF) [file pone.0010318.s007.tif]

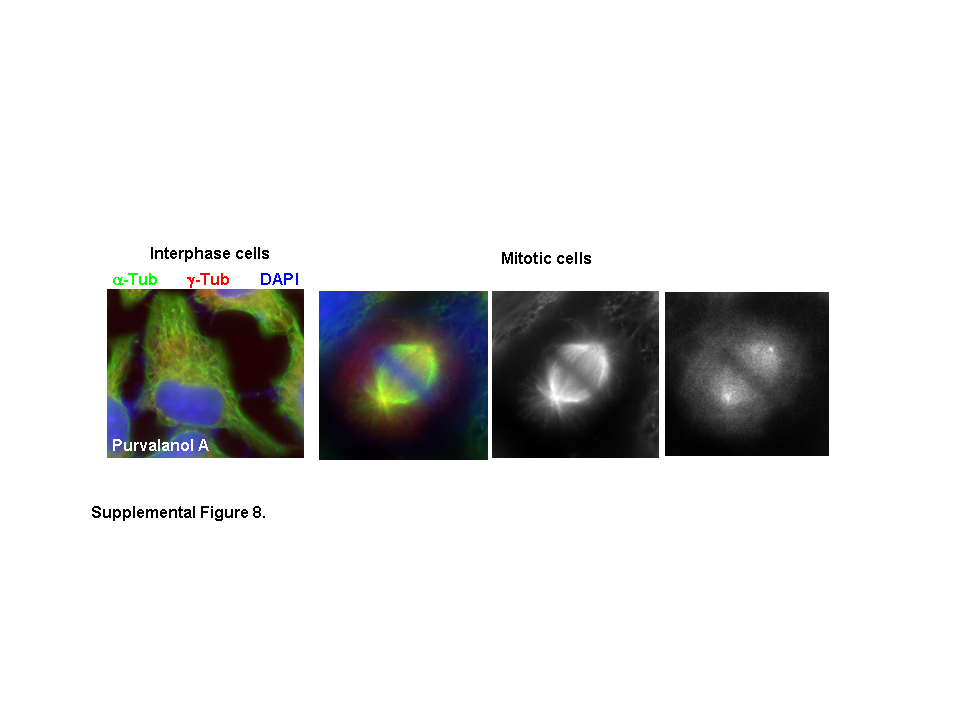

Supplement: Figure S8 — CDK inhibitor does not cause microtubule defects. Representative immunostaining of interphase microtubules and mitotic spindles of HeLa cells treated with Purvalanol A (10 uM) for 1 hour. (0.39 MB TIF) [file pone.0010318.s008.tif]
